# Genetic Landscape of Oral Carcinoma Cuniculatum and its Histological Mimics

**DOI:** 10.1007/s12105-026-01921-3

**Published:** 2026-05-25

**Authors:** Sawako Ono, Yuki Fukawa, Katsutoshi Hirose, Yumiko Hori, Daisuke Motooka, Hiroyuki Harada, Eiichi Morii, Satoru Toyosawa, Naozumi Ishimaru, Hidetaka Yamamoto

**Affiliations:** 1 Department of Pathology and Oncology, Okayama University Graduate School of Medicine, Dentistry and Pharmaceutical Sciences, 2-5-1 Shikata-cho, Kita-ku, Okayama, 700-8558 Japan; 2 Department of Oral Pathology, Graduate School of Medical and Dental Sciences, Institute of Science Tokyo, 1-5-45 Yushima, Bunkyo-ku, Tokyo, 113-8549 Japan; 3 Department of Oral and Maxillofacial Pathology, University of Osaka Graduate School of Dentistry, 1-8 Yamadaoka, Suita, Osaka 565-0871 Japan; 4 Department of Pathology, University of Osaka Graduate School of Medicine, 2-2 Yamadaoka, Suita, Osaka 565-0871 Japan; 5 Department of Central Laboratory and Surgical Pathology, NHO Osaka National Hospital, 2-1-14 Hoenzaka, Chuo-ku, Osaka, 540-0006 Japan; 6 Genome Information Research Center, Research Institute for Microbial Diseases, University of Osaka, 3-1 Yamadaoka, Suita, Osaka 565-0871 Japan; 7 Department of Oral and Maxillofacial Surgical Oncology, Graduate School of Medical and Dental Sciences, Institute of Science Tokyo, 1-5-45 Yushima, Bunkyo- ku, Tokyo, 113-8519 Japan

**Keywords:** Squamous cell carcinoma, Oral squamous cell carcinoma, Carcinoma cuniculatum, TP53, CDKN2A

## Abstract

**Purpose:**

Oral carcinoma cuniculatum (CC) is a rare variant of oral squamous cell carcinoma (OSCC), characterized by a well-differentiated, burrowing invasive pattern and minimal cellular atypia. Its diagnosis is challenging owing to its bland morphology, which makes differentiation from non-neoplastic lesions difficult on biopsy. Despite this, CC often requires distinction from conventional OSCC, even in surgical specimens. Histological overlap with conventional OSCC suggests a continuous spectrum of diseases. To clarify the genetic identity of pure CC, we analyzed 23 cases of OSCC with burrowing invasive patterns and classified them into three groups: CC, conventional OSCC (SCC), and uncertain CC (UCC).

**Methods:**

We retrospectively reviewed 2002 OSCC cases from multicenter archives. From this cohort, 23 cases exhibiting a burrowing invasive pattern characteristic of CC were selected. The cases were classified into the three study groups based on the WHO diagnostic criteria, and clinicopathological features were reviewed. Targeted next-generation sequencing was performed to identify clinically significant genetic alterations, and immunohistochemical staining was also conducted.

**Results:**

The 23 cases were histologically classified into CC (*n* = 8), UCC (*n* = 7), and SCC (*n* = 8) groups. Clinically, the CC group predominantly affected the gingiva with no recurrence or metastasis. Conversely, the SCC group primarily involved the tongue and showed recurrence and metastasis. Genetic alterations were detected in 87.5% (7/8) of CC cases, with low frequencies of *TP53* (12.5%) and *CDKN2A* (25.0%) alterations and higher frequencies of *FAT1* (50.0%), *NOTCH1* (37.5%), *PIK3CA* (37.5%), and *CASP8* (50.0%) alterations. In contrast, the SCC group showed frequent *TP53* (75.0%) and *CDKN2A* (75.0%) alterations, whereas *FAT1* and *NOTCH1* alterations were absent. Consistent with these findings, p53 staining revealed wild-type patterns in the CC group along with a lower Ki-67 labeling index. The UCC group exhibited intermediate clinicopathological and genetic characteristics.

**Conclusion:**

This study provides the first genetic characterization of oral CC, distinct from that of conventional OSCC. This genetic signature may contribute to the biological behavior of CC and offer a potential tool for its differential diagnosis. Further studies with larger cohorts are required to confirm the relationship between CC and conventional OSCC, and to fully elucidate the genetic landscape of CC.

**Supplementary Information:**

The online version contains supplementary material available at 10.1007/s12105-026-01921-3.

## Introduction

Oral squamous cell carcinoma (OSCC) is the most common malignancy of the oral cavity [1, 2]. Carcinoma cuniculatum (CC) is an extremely rare variant of OSCC that is well-differentiated, locally destructive, and non-metastatic [1]. The term “*cuniculus*,” which describes subterranean interconnected networks resembling rabbit burrows, characterizes the hallmark infiltration pattern of this tumor [3, 4]. Aird et al. first described CC of the plantar region in 1954 [3], and Flieger and Owinski reported CC of the oral cavity in 1977 [5]. Histologically, oral CC is defined by a characteristic, unique, burrowing invasive pattern of stratified squamous epithelium with keratin-filled crypts [1]. Notably, significant cytological atypia is consistently absent. Due to its bland morphology and lack of remarkable cytological atypia, the diagnosis of CC remains challenging as it is frequently missed or misdiagnosed, particularly in biopsy specimens [1, 6–10]. Consequently, the preoperative diagnosis is inaccurate in most cases [1, 6–10]. CC is commonly misdiagnosed as other conditions such as reactive hyperplasia, hyperkeratosis, abscess, and osteomyelitis, frequently resulting in treatment delays [7–12]. Genetic analysis may serve as a useful tool for differentiating CC from these conditions [1, 2, 13]. OSCC harbors pathogenic somatic alterations, primarily in genes such as *TP53*, *CDKN2A*, *FAT1*, *NOTCH1*, *PIK3CA*, and *CASP8* [1, 2, 14–16]. Furthermore, specific variants of OSCC have genetic characteristics distinct from those of conventional OSCC variants [17, 18], suggesting that CC may also have characteristics that are different from those of conventional OSCC. To our knowledge, no previous studies have identified genetic alterations in patients with CC. A detailed analysis of the genetic profile of CC may provide a useful tool for its pathological diagnosis and enhance our understanding of its biology.

One obstacle to oral CC research is the difficulty in making a definitive diagnosis, even from surgical specimens [7, 11, 12, 19, 20]. Although CC generally has a very good prognosis, its differentiation from conventional OSCC, which often involves recurrence and metastasis, is challenging, and evaluations are inconsistent even among pathologists [1, 12, 19, 20]. The distinguishing features of CC and conventional OSCC are thought to be their invasion patterns and cellular atypia. However, some conventional OSCC exhibit a similar burrowing infiltrative pattern [19]. Moreover, the evaluation of cellular atypia, such as nuclear pleomorphism, is often inconsistent among pathologists [21]. These facts blur the boundary between CC and conventional OSCC. Based on these characteristics, it has been suggested that CC is a distinct entity that also may exist on a continuous spectrum of diseases with conventional OSCC [6, 19]. Indeed, reports have described a case of CC that included conventional OSCC components [9], a case where a portion of the CC underwent transformation to conventional OSCC upon recurrence [12], a case where CC recurred as conventional OSCC [20], and a case where CC metastases in the lymph node were diagnosed as conventional OSCC [20]. It has also been reported that among previously described CC cases, some do not meet histological diagnostic criteria and cannot be considered pure CC (which we will refer to as uncertain CC [UCC] cases hereinafter) [19]. Thus, to clarify the genetic profile of CC, we considered it necessary to select pure CC from the spectrum of CC to conventional OSCC.

In this study, we evaluated 23 cases of OSCC that exhibited the burrowing invasive pattern characteristic of CC. Based on the WHO histological diagnostic criteria for oral CC [1], the cases were classified into the following three groups: CC (typical CC), SCC (conventional OSCC), and UCC (borderline cases that were histologically difficult to distinguish from the other two). This study aimed to clarify the genetic characteristics of oral CC, as well as histologically similar OSCC. To the best of our knowledge, this is the first study to provide genetic data on CC and other histologically similar diseases.

## Materials and Methods

### Patient Selection and Histological Assessment

We retrospectively reviewed a total of 2002 OSCC cases from the pathology archives of Okayama University Hospital, Institute of Science Tokyo Hospital, Osaka University Dental Hospital, and NHO Osaka National Hospital. From these, 23 cases exhibiting a burrowing invasive pattern were selected. Cases of non-primary or recurrent OSCC were excluded.

Resected tissue specimens were fixed in 10% neutral-buffered formalin and embedded in paraffin. The resulting formalin-fixed, paraffin-embedded (FFPE) tissue specimens were sectioned into 4-µm-thick serial sections and subjected to hematoxylin and eosin (H&E) and immunohistochemical stainings. Three experienced oral and maxillofacial pathologists (SO, YF, and KH) from different institutions independently reviewed the 23 OSCC cases. The classification was based on the essential diagnostic criteria for CC as defined by the WHO Classification of Head and Neck Tumors, 5th edition: well-differentiated, keratin-containing crypts, no more than mild atypia, and a labyrinthine, burrowing, and cohesive invasive pattern [1]. In addition, the pathologists assessed each case according to the desirable diagnostic criteria (keratin microabscesses, stromal neutrophils, microsequestra, bone invasion at mucoperiosteal sites) and the presence of epithelial changes at the tumor margins. After independent reviews, a final consensus diagnosis was reached for each case through discussion.

Based on this consensus, cases that fulfilled all essential criteria with unanimous agreement among the three pathologists were classified into the CC group. In contrast, cases that fulfilled some essential criteria, but with some interobserver disagreement regarding a definitive diagnosis of CC, were classified into the UCC group. Finally, cases that, despite potentially fulfilling some essential criteria, were unanimously determined not to be CC on the overall assessment were classified into the SCC group.

## Immunohistochemistry

Immunohistochemical staining was performed on paraffin sections using the Bond III automated stainer (Leica Biosystems, Deer Park, IL, USA). Primary antibodies included anti-p53 (clone Pab1801, Cat# sc-98, Santa Cruz Biotechnology, Dallas, TX, USA) and anti-Ki-67 (clone MM1, Cat# NCL-L-Ki-67-MM1, Leica Biosystems). The immunohistochemical staining pattern of p53 was classified as either wild-type or abnormal, according to previously described criteria [22].

## Molecular Analysis

Next-generation sequencing was performed using the SureSelect Cancer CGP Assay (Agilent Technologies, Santa Clara, CA, USA) as previously described [18]. The DNA-based cancer gene panel was designed to detect genetic alterations of clinical significance, including single-nucleotide variants (SNVs) and small insertions/deletions (Indels) in 679 genes and copy number variations (CNVs) in 32 genes. The investigated genes are listed in Supplementary Table 1. Two pathologists (SO and KH) identified FFPE blocks of the analyzed cases with > 15% tumor content. Genomic DNA was extracted from FFPE tissues using a QIAamp DNA FFPE Tissue Kit (Qiagen, Valencia, CA, USA) according to the manufacturer’s instructions. Sequence libraries were prepared using the custom SureSelect Low-Input Target Enrichment System (Agilent Technologies) and sequenced using Illumina MiSeq (Illumina, San Diego, CA, USA). Alissa Reporter ver1.3.3 (Agilent Technologies) was used for variant calling and for calculating tumor mutational burden (TMB) and microsatellite instability (MSI). Intron DNA, non-coding DNA, variant allele frequency < 5%, and minor allele frequency > 0.001 based on the gnomAD database were excluded.

The pathogenicity of the detected variants (SNVs/Indels) was systematically classified. First, the variants were annotated against the ClinVar and InterVar databases. Those classified by the databases as “Pathogenic” or “Likely pathogenic” were categorized as “Pathogenic mutation” in the Results section. Variants that did not meet these criteria and those labeled as “uncertain significance” in the ClinVar or InterVar databases were processed for further analysis using in silico functional prediction tools. Variants were classified as “variant of uncertain significance” (VUS) in the Results section if they were predicted to be deleterious by at least one of the following tools: SIFT (https://sift.bii.a-star.edu.sg/), PolyPhen-2 (http://genetics.bwh.harvard.edu/pph2/), and MutationTaster (https://www.mutationtaster.org/). These molecular analyses were performed for the purposes of this study. Three molecular pathologists (YF, KH, and YH) and a bioinformatician (DM) evaluated the interpretation of the results.

## Statistical Analyses

Data are expressed as means ± standard deviations. Statistical analyses were performed and graphs were created using Microsoft Excel and GraphPad Prism (version 10; La Jolla, CA, USA). Statistical significance was set at a* p*-value < 0.05. Data were tested using Fisher’s exact test and Tukey’s multiple comparison test.

## Results

### Case Classification

**Fig. 1:**
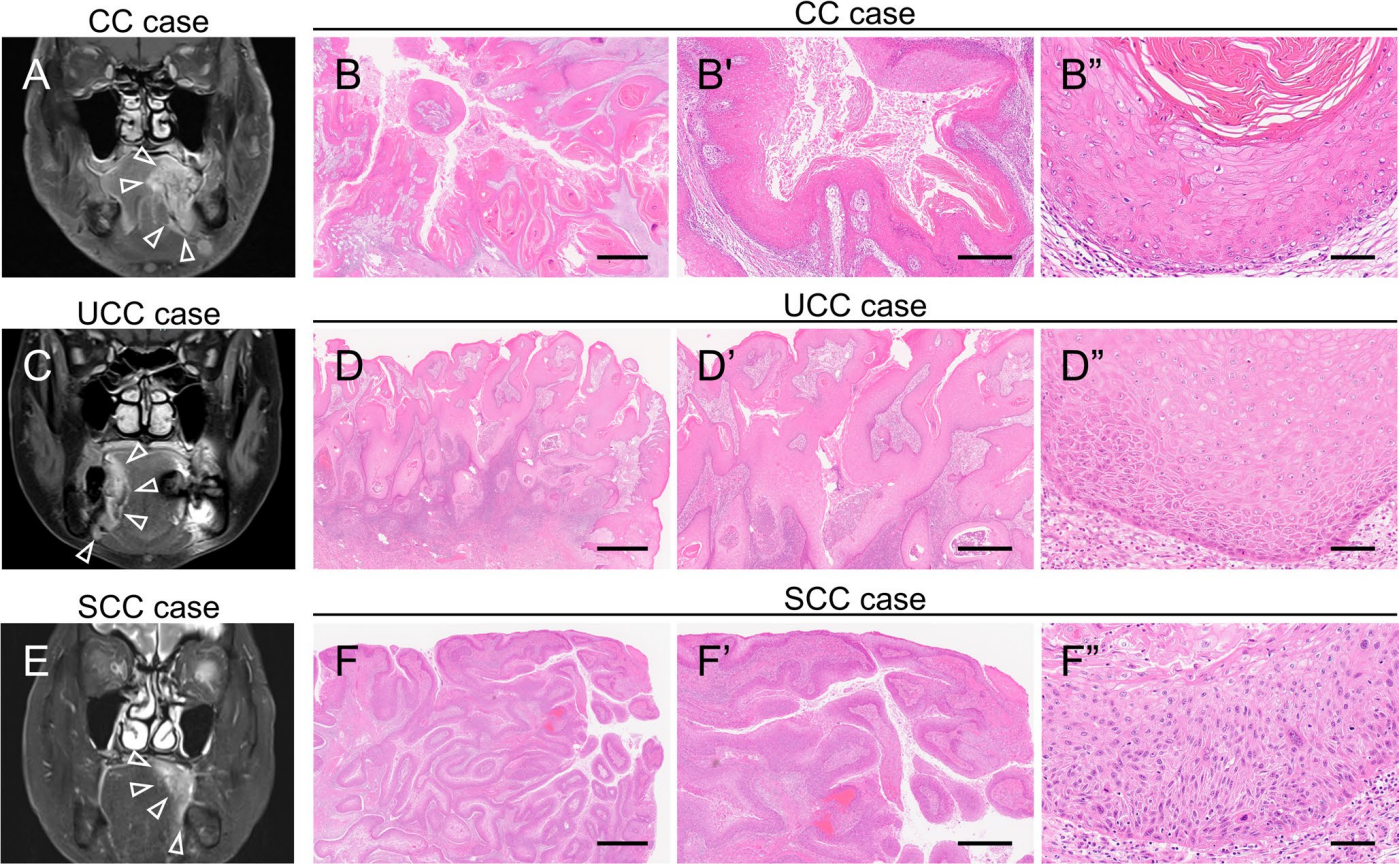
MRI and histological findings of the CC, UCC, and SCC groups. **A–F**: Representative preoperative T2-weighted MRI image (**A**,** C**,** E**) and H&E stainings (**B**,** D**,** F**). Panels A, C, and E show MRI images of the CC, UCC, and SCC groups, respectively (arrowheads indicating the location of the tumor). Panels B–B”, D–D”, and F–F” show H&E images of the CC, UCC, and SCC groups, respectively (**B**,** D**,** F**: low magnification; **B’**,** D’**,** F**’: intermediate magnification; **B”**,** D”**,** F”**: high magnification). The UCC case exhibits several features suggestive of CC but shows incomplete labyrinthine architecture and an inadequate amount of intracryptal keratin (**D**). The SCC case exhibits a burrowing invasive pattern with marked cytological atypia that is greater than mild (**F**). Scale bars: B, D, F = 2500 μm; B’, D’, F’ = 1000 μm; B”, D”, F” = 100 μm. *CC* carcinoma cuniculatum, *H&E* hematoxylin and eosin, *MRI* magnetic resonance imaging, *SCC* squamous cell carcinoma, *UCC* uncertain carcinoma cuniculatum

We retrospectively investigated 23 cases of OSCC characterized by burrowing invasive growth. These cases were histologically classified into CC and SCC groups based on the essential diagnostic criteria for CC according to the WHO classification [1]. Cases that could not be clearly classified as CC or conventional OSCC were conveniently assigned to the UCC group in this study. On magnetic resonance imaging, lesions from all three groups exhibited an endophytic growth pattern (Fig. 1A, C and E). Consistent with its classification, the CC group exhibited all essential histological features of typical CC, including keratin-containing crypts, no more than mild atypia, a labyrinthine, burrowing, cohesive invasive pattern, and being well-differentiated (Fig. 1B–Bʺ). The UCC group exhibited several features suggestive of CC, but these were ultimately classified as uncertain owing to interobserver disagreement. The points of contention included the degree of cellular atypia, the incompleteness of the labyrinthine architecture with unclear continuity (in 5/7 cases), and the inadequate amount of intracryptal keratin (in 3/7 cases) (Fig. 1D–Dʺ). The SCC group also met some of the essential criteria for CC, including burrowing growth patterns. However, the cases in this group were ultimately classified as SCC due to the presence of features inconsistent with a CC diagnosis, such as cytologic atypia greater than mild and a non-cohesive invasive front, making them more characteristic of conventional OSCC (Fig. 1F–Fʺ). Other histological features (including desirable diagnostic criteria and the presence of epithelial changes at the tumor margins) among the three groups are summarized in Supplementary Fig. 1.

**Fig. 2:**
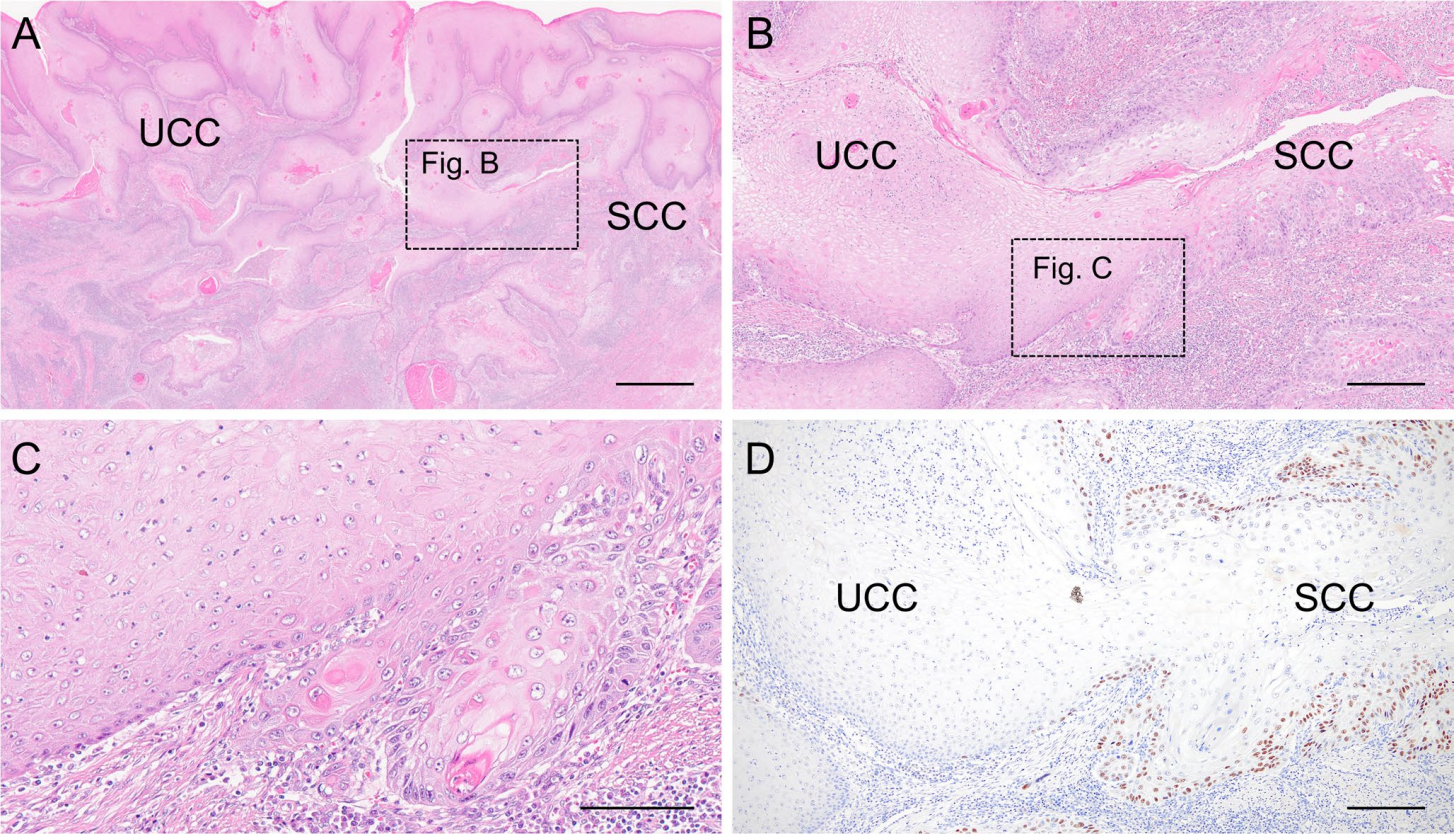
Histopathological findings of coexisting UCC and SCC components. **A-C**: Representative H&E images of a case with coexisting UCC and SCC components (**A**: low magnification; **B**: intermediate magnification; **C**: high magnification). The left side shows the UCC component with incomplete labyrinthine architecture and an inadequate amount of intracryptal keratin. The right side shows the SCC component with marked cytological atypia and conventional invasive features (**B** and **C**: higher magnifications of the black dotted boxes in A and B, respectively). **D**: p53 immunostaining at the boundary between UCC and SCC, corresponding to the H&E-stained image shown in B. An abnormal p53 staining pattern is observed in the SCC region. Scale bars: A = 2500 μm; B, D = 1000 μm; C = 200 μm. *H&E* hematoxylin and eosin, *SCC* squamous cell carcinoma, *UCC* uncertain carcinoma cuniculatum

Of the 23 cases, one was initially considered for the UCC group because of an incomplete labyrinthine architecture and inadequate intracryptal keratin (Fig. 2A–C). However, because this case also showed components of conventional OSCC with marked cellular atypia and an abnormal p53 immunostaining pattern, it was ultimately classified as the SCC group (Fig. 2C and D).

### Clinical Validation

To verify that the characteristics of the CC group were consistent with the known clinical profile of CC and distinct from those of the other groups, we compared the clinical characteristics of the three histologically categorized groups (Table 1). Overall, the median age of the 23 patients was 74 (range, 44–88) years, with 15 (65.2%) male and 8 (34.8%) female patients. The median ages of patients in the CC, UCC, and SCC groups were 71, 78, and 74 years, respectively. The most common primary site was the gingiva (11/23 cases, 47.8%), followed by the tongue (8/23 cases, 34.8%). Gingival tumors were predominant in the CC and UCC groups (6/8 cases, 75.0% and 4/7 cases, 57.1%, respectively), whereas tongue tumors were most frequent in the SCC group (4/8 cases, 50.0%). Regarding tumor stage in the entire cohort, early-stage (T1–T2) tumors accounted for 9 cases (39.1%), and advanced-stage (T3–T4) tumors accounted for 14 cases (60.9%). Advanced T stages (T3–T4) were most frequent in the CC group (7/8 cases, 87.5%), followed by the SCC group (5/8 cases, 62.5%), whereas early stages (T1–T2) were more common in the UCC group (5/7 cases, 71.4%). Regarding nodal metastasis status, 20 cases (87.0%) were negative, and 3 cases (13.0%) were positive. All positive cases were confined to the SCC group (3/8 cases, 37.5% of the SCC group). Recurrence or postoperative distant metastasis was observed in 2 cases (8.7%), with both cases occurring in the SCC group (2/8 cases, 25.0% of the SCC group). No recurrence or metastasis was observed in either the CC or UCC group.

**Table 1:** Clinical characteristics of oral squamous cell carcinomas with a burrowing invasive pattern

Factors	CC (*n* = 8)	UCC (*n* = 7)	SCC (*n* = 8)	Total (*n* = 23)
*Age (years)*				
Median (range)	71 (60–82)	78 (45–88)	74 (44–80)	74 (44–88)
*Sex*				
Male	5 (62.5%)	4 (57.1%)	6 (75.0%)	15 (65.2%)
Female	3 (37.5%)	3 (42.9%)	2 (25.0%)	8 (34.8%)
*Primary tumor site*				
Gingiva	6 (75.0%)	4 (57.1%)	1 (12.5%)	11 (47.8%)
Tongue	2 (25.0%)	2 (28.6%)	4 (50.0%)	8 (34.8%)
Floor of mouth	0 (0%)	0 (0%)	2 (25.0%)	2 (8.7%)
Buccal mucosa	0 (0%)	0 (0%)	1 (12.5%)	1 (4.3%)
Hard palate	0 (0%)	1 (14.3%)	0 (0%)	1 (4.3%)
*T stage*				
T1–T2	1 (12.5%)	5 (71.4%)	3 (37.5%)	9 (39.1%)
T3–T4	7 (87.5%)	2 (28.6%)	5 (62.5%)	14 (60.9%)
*Lymphatic metastasis*				
Negative	8 (100%)	7 (100%)	5 (62.5%)	20 (87.0%)
Positive	0 (0%)	0 (0%)	3 (37.5%)	3 (13.0%)
*Follow-up (months)*				
Median (range)	43 (5–79)	30 (8–64)	13 (1–107)	29 (1–107)
*Events during follow-up*				
Postoperative recurrence or metastasis	0 (0%)	0 (0%)	2 (25.0%)	2 (8.7%)

CC, carcinoma cuniculatum; SCC, squamous cell carcinoma; UCC, uncertain carcinoma cuniculatum

The clinical characteristics of the CC group, such as age, sex, tumor site, and absence of metastasis, were consistent with those reported in previous studies [1, 8, 23, 24]. These findings suggest that the histology-based categorization of OSCC exhibiting a burrowing infiltrative pattern may effectively distinguish pure CC from conventional OSCC. The UCC group may represent a clinical entity with intermediate characteristics between those of the CC and SCC groups.

### Msolecular genetic analysis

**Fig. 3:**
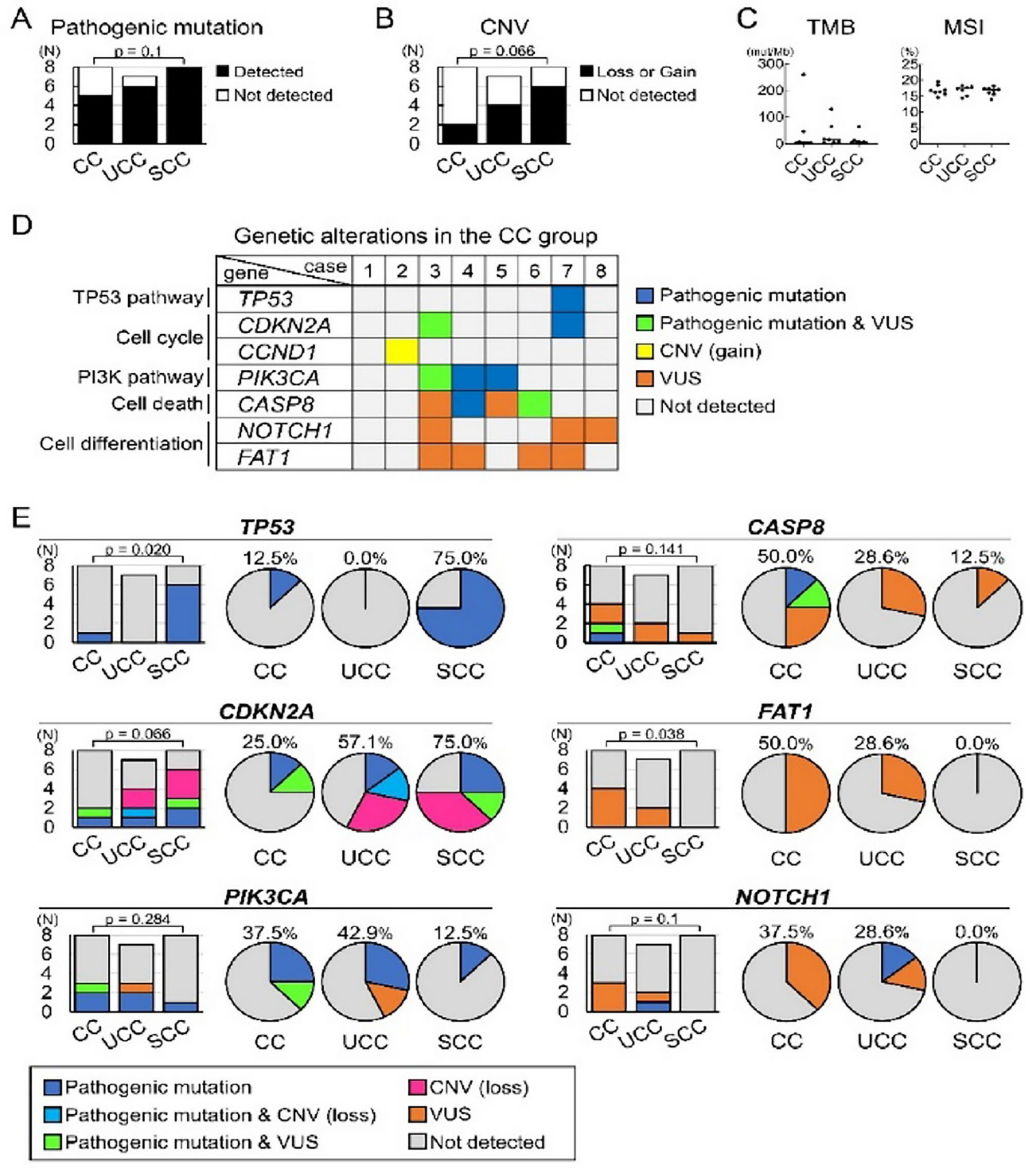
Genetic profiles of the CC, UCC, and SCC groups. **A**: Proportion of cases harboring at least one pathogenic mutation (SNVs or Indels) among the CC, UCC, and SCC groups. **B**: Proportion of cases with CNVs in the three groups. **A**,** B**: p-values shown above bar plots indicate comparisons of alteration frequencies between the CC and SCC groups (Fisher’s exact test). **C**: TMB and MSI distributions in the three groups (each data point represents one case) (Tukey’s multiple comparison test). **D**: Summary of genetic alterations in the CC group, focusing on key genes implicated in oral SCC pathogenesis. **E**: Proportion of cases with alterations in each key gene. Stacked bar plots show the number of cases with any alteration per group, and pie charts show the percentage of altered cases in each group. VUS mutations are genetic mutations predicted to be deleterious based on in silico prediction tools. p-values shown above bar plots indicate comparisons of the alteration frequencies between the CC and SCC groups (Fisher’s exact test). *CASP8* caspase 8, *CC* carcinoma cuniculatum, *CCND1*, cyclin D1, *CDKN2A* cyclin dependent kinase inhibitor 2 A, *CNV* copy number variation, *FAT1* FAT atypical cadherin 1, *indel* insertion/deletion, *MSI* microsatellite instability, *NOTCH1* Notch receptor 1, *PI3K* phosphoinositide 3-kinase, *PIK3CA* phosphoinositide 3-kinase catalytic subunit alpha, *SCC* squamous cell carcinoma, *SNV* single-nucleotide variant, *TMB* tumor mutational burden, *TP53* tumor protein p53, *UCC* uncertain carcinoma cuniculatum, *VUS* variant of uncertain significance

We investigated the genetic characteristics of the CC group, as well as the UCC and SCC groups. Overall, genetic alterations, including both pathogenic mutations (SNVs/Indels) and CNVs (gain/loss), were detected in 75.0% (6/8 cases), 100% (7/7 cases), and 100% (8/8 cases) of the CC, UCC, and SCC groups, respectively (Fig. 3A and B). The most frequently altered genes across all groups were *CDKN2A* (12/23 cases), *TP53* (7/23 cases), and *PIK3CA* (6/23 cases). The frequencies of pathogenic mutations (*p* = 0.10) or CNVs (*p* = 0.066) did not statistically significantly differ between the CC and SCC groups (Fig. 3A and B). No significant differences in TMB were observed among the three groups, with overall low scores (Fig. 3C). MSI scores were likewise low, and all cases were microsatellite stable (Fig. 3C). Details of the pathogenic mutations and CNVs are shown in Supplementary Fig. 2.

Next, we sought to elucidate the detailed genetic characteristics of the CC group by focusing on key genes implicated in OSCC pathogenesis [1, 2, 14–16] (Fig. 3D). Genetic alterations, including pathogenic and VUS mutations and CNVs, were detected in 87.5% (7/8 cases) of patients in the CC group (Fig. 3D). VUS mutations are genetic mutations predicted to be deleterious by in silico prediction tools. Two major causal genes in OSCC, *TP53* and *CDKN2A*, were detected in only a small number of patients in the CC group (Fig. 3D). In contrast, mutations in *PIK3CA* (related to the phosphoinositide 3-kinase [PI3K] pathway), *CASP8* (related to cell death), and *FAT1* and *NOTCH1* (related to cell differentiation) were detected in approximately half of the CC group [2, 25, 26]. These findings suggest that CC and conventional OSCC may have distinct genetic profiles, and the genetic analysis of CC could provide a useful tool for differential diagnosis, especially for non-neoplastic epithelial lesions.

We investigated whether the genetic profile of the CC group differed from that of the other histologically similar disorders (Fig. 3E). A difference was observed in *TP53* mutations, which were significantly more frequent in the SCC group (6/8 cases, 75.0%) than in the CC (1/8 cases, 12.5%) group (*p* = 0.020). Similarly, alterations in *CDKN2A* (including pathogenic/VUS mutations or CNV loss) showed a trend towards a higher frequency in the SCC group (6/8 cases, 75.0%) than in the CC group (2/8 cases, 25.0%) (*p* = 0.066). Conversely, *FAT1* mutations, all of which were classified as VUS, were significantly more prevalent in the CC (4/8 cases, 50.0%) and UCC (2/7 cases, 28.6%) groups and were entirely absent in the SCC group (0/8 cases, 0.0%; *p* = 0.038 for CC vs. SCC). All *FAT1* mutations in the CC group were stopgain, whereas those in the UCC group were nonsynonymous. A similar pattern was observed for *NOTCH1* mutations, which were detected only in the CC (3/8 cases, 37.5%) and UCC (2/7 cases, 28.6%) groups. Alterations in *PIK3CA* and *CASP8* levels were detected in all three groups, without significant differences between the CC and SCC groups.

Collectively, these results revealed that the genetic profile of the CC group was different from that of the SCC group (Fig. 3E). The SCC group was characterized by a high frequency of alterations in tumor suppressor genes such as *TP53* and *CDKN2A*, consistent with previous reports on OSCC [1, 2, 14–16]. In contrast, the CC group showed alterations in *FAT1* and *NOTCH1*, which were absent in the SCC group. The UCC group exhibited genetic characteristics intermediate between those of the CC and SCC groups (Fig. 3E).

### Immunohistochemical finding

**Fig. 4:**
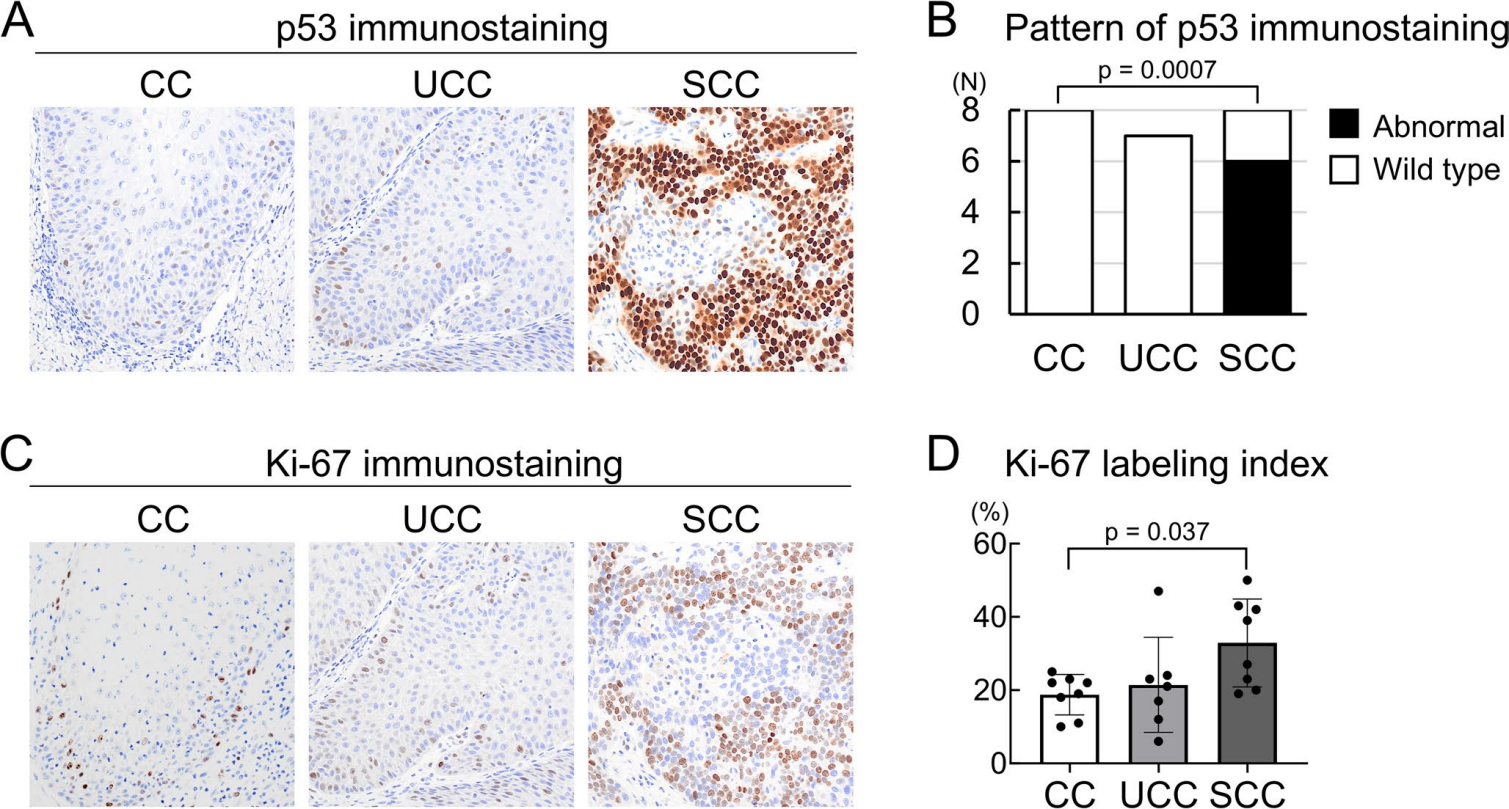
p53 and Ki-67 immunohistochemical profiles of the CC, UCC, and SCC groups. **A**: Representative images of p53 immunostainings of the CC, UCC, and SCC groups. **B**: Proportion of cases with p53 immunostaining patterns across the three groups. p-values shown above bar plots indicate comparisons of the staining patterns between the CC and SCC groups (Fisher’s exact test). **C**: Representative images of Ki-67 immunostainings of the CC, UCC, and SCC groups. **D**: Ki-67 labeling index distributions for the three groups (each data point represents one case). p-values show the comparison among the three groups (Tukey’s multiple comparison test). *CC* carcinoma cuniculatum, *SCC* squamous cell carcinoma, *UCC* uncertain carcinoma cuniculatum

Both *TP53* and *CDKN2A* are major tumor suppressor genes in OSCC, and *CDKN2A* is involved in cell cycle regulation [1, 2, 27, 28]. We hypothesized that these genetic alterations might be involved in the phenotypes and biological behaviors of the CC group, as well as the UCC and SCC groups. Therefore, we performed immunohistochemistry for p53 and Ki-67 to evaluate differences among the three groups. Immunohistochemical examination of p53 revealed a wild-type staining pattern in the CC and UCC groups and an abnormal staining pattern in the SCC group (Fig. 4A). The wild-type staining pattern in the CC group was significantly different from that in the SCC group (*p* = 0.0007), which was consistent with the results of the genetic analysis (Figs. 3E and 4B). The Ki-67 labeling index was 18.8 ± 5.2% in the CC group, 21.4 ± 12.0% in the UCC group, and 32.9 ± 11.2% in the SCC group. The CC group showed a significantly lower proliferation rate than the SCC group (*p* = 0.037) (Fig. 4C and D).

## Discussion

In the present study, we conducted the first genetic analysis of clinically and histologically confirmed CC of the oral cavity. Genetic alterations were detected in almost all CC cases (Fig. 3D). CC cases were characterized by a low frequency of mutations in two major OSCC driver genes, *TP53* and *CDKN2A*, coupled with a high frequency of alterations in other key genes, such as *FAT1* and *NOTCH1*. Thus, CC may have genetic profiles distinct from those of conventional OSCC, and genetic analysis of CC may provide a useful tool for differential diagnosis, especially for non-neoplastic lesions.

Oral CC is an extremely rare variant of OSCC that is histologically characterized by a well-differentiated appearance and burrowing-like invasive pattern. Clinically, it has an excellent prognosis, marked by rare recurrence and the absence of metastasis [1]. Typical clinical features include an onset in patients aged 60–70 years, a slight male predominance, and a predilection for the gingiva as the most common site, followed by the tongue [1, 8, 23, 24]. Our CC group had a mean age of approximately 70 years and a slight male predominance (Table 1). The most common site was the gingiva, followed by the tongue. Furthermore, the CC group showed no lymph node metastases or recurrence. These results support the notion that our CC group represents cases of pure oral CC.

In our CC group, mutations were detected in *TP53* (12.5%), *CDKN2A* (SNVs/Indels, 25%; CNVs, 0.0%), *FAT1* (50.0%), *NOTCH1* (37.5%), *PIK3CA* (37.5%), and *CASP8* (50.0%) (Fig. 3E). For comparison, the mutation frequencies in general OSCC cohorts, including data from previous reports and the Cancer Genome Atlas (TCGA) database, are as follows: *TP53* (61.7–70.4%), *CDKN2A* (SNVs/Indels, 12.8–26.0%; CNVs, 14.9–74.0%), *FAT1* (9.1–30.0%), *NOTCH1* (3.2–25.5%), *PIK3CA* (10.6–16.8%), and *CASP8* (10.0–13.9%) [14–16]. Compared with these general OSCC cohorts, our CC group exhibited notably lower mutation frequencies in *TP53* and lower *CDKN2A* CNVs, along with higher frequencies in *FAT1*, *NOTCH1*, *PIK3CA*, and *CASP8*. Furthermore, verrucous carcinoma, a rare OSCC variant characterized by exophytic growth and well-differentiated histology, lacks genetic alterations in *TP53*, *CDKN2A*, *FAT1*, and *NOTCH1* [17], a finding which supports the distinct genetic profile of CC. Further investigations are warranted to determine whether these gene mutations contribute to the specific biology of CC.

A combination of mutations in these four genes (*FAT1*, *NOTCH1*, *PIK3CA*, and *CASP8*) was found in 75.0% (6/8 cases) of the patients in the CC group (Fig. 3D). This suggests that analyzing these mutations might be a useful tool for distinguishing CC from non-neoplastic epithelial lesions, especially in biopsy samples. Furthermore, the high frequency of *PIK3CA* mutations is clinically significant as it is a therapeutic target. PI3K inhibitors, which have proven antitumor activity, may expand potential treatment options for this specific OSCC variant [29, 30].

All *FAT1* mutations detected in the present study were classified as VUS using database annotation and in silico prediction (Fig. 3D and E). Under physiological conditions, FAT1 regulates actin dynamics associated with cell adhesion and polarity, contributes to development and organ formation, and modulates mitochondrial function [31, 32]. However, given the large size of *FAT1* mRNA and the protein it encodes, clarifying the biological function of FAT1 remains challenging [31, 32]. Although *FAT1* is among the most frequently mutated genes across human cancers, it lacks clearly defined mutational hotspots, and the functional consequences of individual *FAT1* variants are often unclear [31]. Consistent with this, most *FAT1* variants are categorized as “uncertain significance” in the ClinVar database. While *FAT1* mutations have been implicated in the initiation and progression of OSCC [2, 15, 31], further investigations are warranted to clarify their contribution to CC.

Both *TP53* and *CDKN2A* are major tumor suppressor genes in OSCC. *TP53* is activated in response to DNA damage and is mutated in most common human malignancies [2, 27], whereas *CDKN2A* is involved in cell cycle regulation [2, 28]. Furthermore, mutations in these two genes often co-occur and are correlated with poor prognosis, such as lower overall survival rates and treatment resistance [2, 27, 28]. Consistent with these genetic findings, our CC group exhibited a wild-type staining pattern for p53 and a low Ki-67 labeling index (Fig. 4B and D). Although a few previously reported cases of CC showed both wild-type and abnormal p53 staining patterns [6, 8, 11], these cases lacked genetic analysis for confirmation. The low Ki-67 labeling index (< 5–15%) observed in previous CC cases is compatible with that in our CC group [8, 11, 33]. The characteristic wild-type p53 staining and low Ki-67 labeling index of CC may make it difficult to distinguish CC from non-neoplastic epithelial lesions, particularly in biopsies. In contrast, the SCC group showed an abnormal p53 staining pattern and a high Ki-67 labeling index (Fig. 4B and D). Therefore, as supported by our genetic analysis, p53 staining pattern and Ki-67 index may serve as valuable ancillary tools for distinguishing between CC and histologically similar OSCC cases. Furthermore, given the multifocal nature of OSCC, previously reported cases in which primary CC metastasized as, or recurred as conventional OSCC [12, 20] may have harbored a coexisting SCC component or, alternatively, possessed intrinsic potential for progression toward an SCC phenotype.

Although their biological behaviors differ, the histological boundary between CC and conventional OSCC is often ambiguous. Based on histological features, it has been suggested that CC is a distinct entity that also may exist on a continuous spectrum of diseases with conventional OSCC [6, 19]. In the present study, we categorized cases that were difficult to classify as either CC or conventional OSCC into the UCC group. Our results demonstrated that the UCC group exhibited intermediate characteristics between the CC and SCC groups across clinical, pathological, and genetic profiles. This intermediate state raises two possibilities.

The first possibility is that the UCC group does not represent a distinct biological entity, but rather a heterogeneous mixture of the CC and SCC groups. This is plausible given that the UCC designation arose from a lack of diagnostic consensus among the three pathologists who used the WHO criteria. Alternative classification methods, perhaps those incorporating additional molecular markers, may resolve these indeterminate cases in the CC or SCC categories.

Second, OSCC with a burrowing invasive pattern may represent a continuous biological spectrum ranging from CC to SCC, with UCC as an intermediate phase, as previously suggested [6, 19]. Our findings partially support this notion, as one of our cases showed histological coexistence of UCC and SCC (Fig. 2). In fact, CC cases with SCC components have been reported [9]. It is unclear whether the histological features change from CC to SCC over time or whether aggressive transformation occurs because of the accumulation of genetic alterations. As an example of transformation, the acquisition of *TP53* or *CDKN2A* mutations in addition to initial *KRAS* or *EGFR* mutations drives the aggressive transformation in inverted papillomas of the nasal cavity [34]. A similar mechanism involving the sequential accumulation of genetic alterations might underlie the progression of CC to SCC in a UCC-like state. However, further investigations are required to validate this hypothesis.

Interpretation of the UCC group in the present study requires caution. While we provisionally categorized a subset of OSCC cases as UCC—with the aim of clarifying the genetic characteristics of pure CC—this classification may be challenging for both research groups and practicing pathologists. This difficulty arises because UCC comprises a diagnostically ambiguous category; even within our group, despite some pathologists diagnosing these cases as CC, we could not reach unanimous consensus on their classification. The UCC group exhibited characteristics intermediate between the CC and SCC groups. Nevertheless, the absence of *TP53* mutations (Fig. 3E), a low Ki-67 labeling index (Fig. 4D), and lack of metastasis or recurrence (Table 1) suggest that the UCC group may be more closely related to the CC group than to the SCC group. For research and diagnosis, a two-category classification of CC and SCC is preferable to the three-category classification used in this study. The p53 staining pattern and Ki-67 index may serve as valuable auxiliary tools for categorizing UCC as either CC or conventional OSCC.

This study has several limitations. First, our analysis intentionally focused on OSCCs with a burrowing invasive pattern, which is characteristic of CC. This selection criterion, which was necessary for our study design, may have introduced bias. Consequently, our SCC control group is not fully representative of conventional OSCCs. Therefore, the differences observed in the present study should be interpreted as a comparison between CC and a histologically similar subset of OSCC rather than between CC and OSCC in general. However, this targeted approach is unlikely to distort the intrinsic clinicopathological and genetic characteristics of CC. Second, the statistical power of this study was inherently limited by its small sample size, which is an unavoidable consequence of the rarity of CC. Therefore, our findings, particularly regarding the genetic landscape of CC and its effects on tumor biology, require validation in larger multi-institutional cohort studies.

## Conclusions

This study provided the first comprehensive description of the genetic characteristics of oral CC. We demonstrated that pure CC possesses a genetic profile distinct from that of conventional OSCC, characterized by a low frequency of *TP53* and *CDKN2A* alterations coupled with a high frequency of mutations in key genes. This genetic signature may contribute to the distinct biological behavior of CC and offer a potential tool for differential diagnosis, particularly for non-neoplastic lesions. While our findings suggest that CC and conventional OSCC may represent a continuous spectrum of diseases, further studies with larger cohorts are required to confirm this relationship and fully elucidate the genetic landscape of CC.

## Supplementary Information

Below is the link to the electronic supplementary material.

Supplementary Material 1

Supplementary Material 2

## Data Availability

The surgical materials and datasets analyzed in this study are available from the corresponding author upon request.

## Abbreviations

CASP8: Caspase 8
CC: Carcinoma cuniculatum
CCND1: Cyclin D1
CDKN2A: Cyclin dependent kinase inhibitor 2 A
CNV: Copy number variation
EGFR: Epidermal growth factor receptor
FAT1: FAT atypical cadherin 1
FFPE: Formalin-fixed, paraffin-embedded
H&E: Hematoxylin and eosin
Indel: Insertion/deletion
KRAS: Kirsten rat sarcoma viral oncogene homolog
MRI: Magnetic resonance imaging
MSI: Microsatellite instability
NOTCH1: Notch receptor 1
OSCC: Oral squamous cell carcinoma
PI3K: Phosphoinositide 3-kinase
SCC: Squamous cell carcinoma
SNV: Single-nucleotide variant
TCGA: The Cancer Genome Atlas
TMB: Tumor mutational burden
TP53: Tumor protein p53
UCC: Uncertain carcinoma cuniculatum
