# Supplementary material for: Genetic Landscape of Oral Carcinoma Cuniculatum and its Histological Mimics
Source: Head Neck Pathol. 2026 May 25;20(1):48. doi: 10.1007/s12105-026-01921-3 (PMC13201809; doi:10.1007/s12105-026-01921-3)
Supplement: Supplementary file 1 — Supplementary Material 1 [file 12105_2026_1921_MOESM1_ESM.docx]

# Supplementary Information

***Head and Neck Pathology***

**Title**

Genetic Landscape of Oral Carcinoma Cuniculatum and Its Histological Mimics

**Authors**

Sawako Ono, Yuki Fukawa, Katsutoshi Hirose, Yumiko Hori, Daisuke Motooka, Hiroyuki Harada, Eiichi Morii, Satoru Toyosawa, Naozumi Ishimaru, Hidetaka Yamamoto

**Corresponding author**

Katsutoshi Hirose

Department of Oral and Maxillofacial Pathology, University of Osaka Graduate School of Dentistry, 1-8 Yamadaoka, Suita, Osaka 565-0871, Japan

E-mail: hirose.katsutoshi.dent@osaka-u.ac.jp

Tel: +81-6-6879-2892; Fax: +81-6-6879-2895

ORCID: 0000-0003-4148-1106


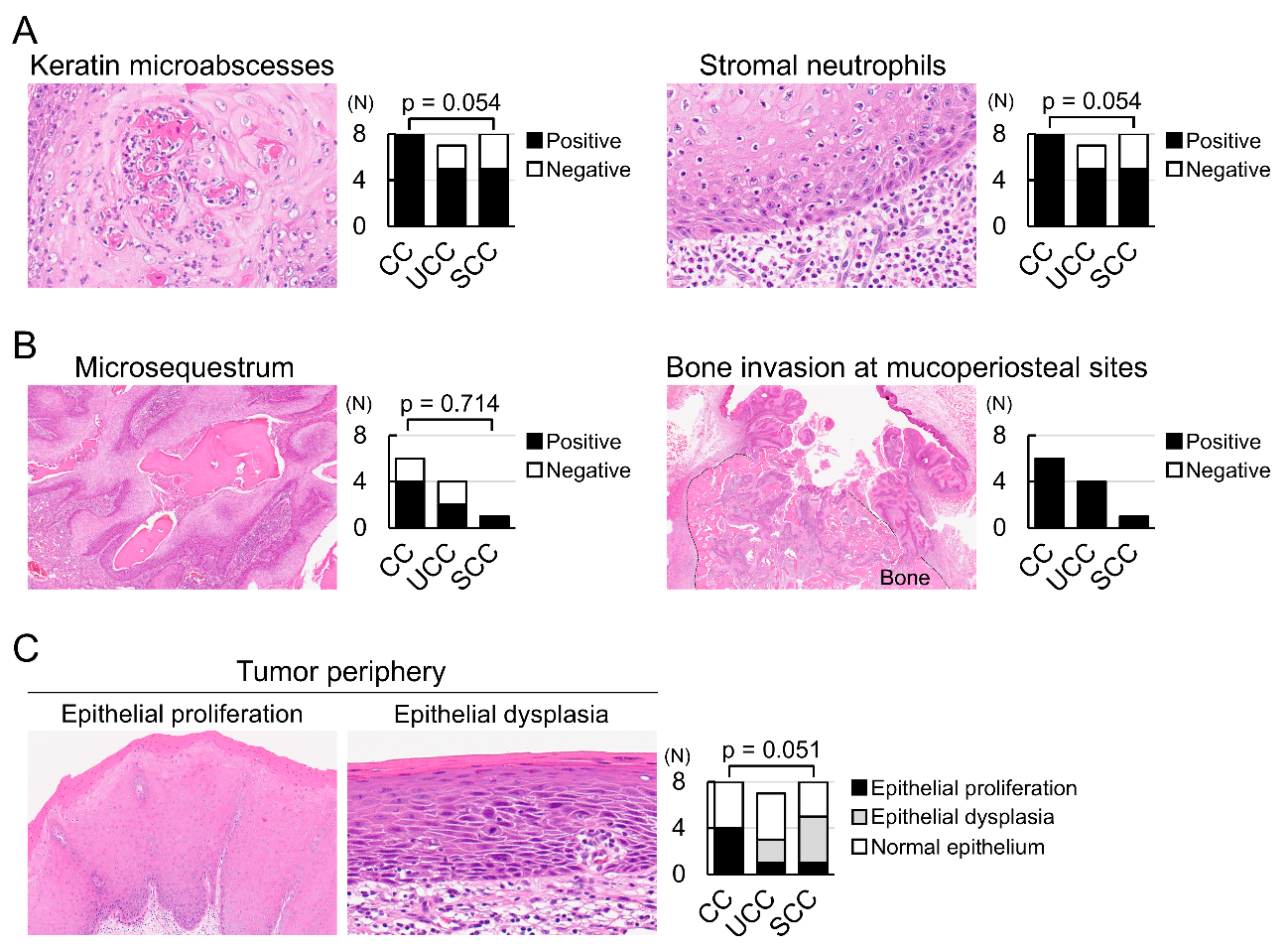


**Supplementary Fig. 1** Summary of the histological features of the CC, UCC, and SCC groups. **A, B:** Representative H&E images and proportions of cases meeting the desired criteria of the CC, UCC, and SCC groups. p-values shown above bar plots indicate comparisons of the proportions of desirable criteria between the CC and SCC groups (Fisher’s exact test). **C:** Representative H&E images and proportions of epithelial changes at the tumor margins in CC, UCC, and SCC. p-values shown above bar plots indicate comparisons of the proportions of epithelial changes at the tumor margins between the CC and SCC groups (Fisher’s exact test). *CC* carcinoma cuniculatum, *H&E* hematoxylin and eosin, *SCC* squamous cell carcinoma, *UCC* uncertain carcinoma cuniculatum


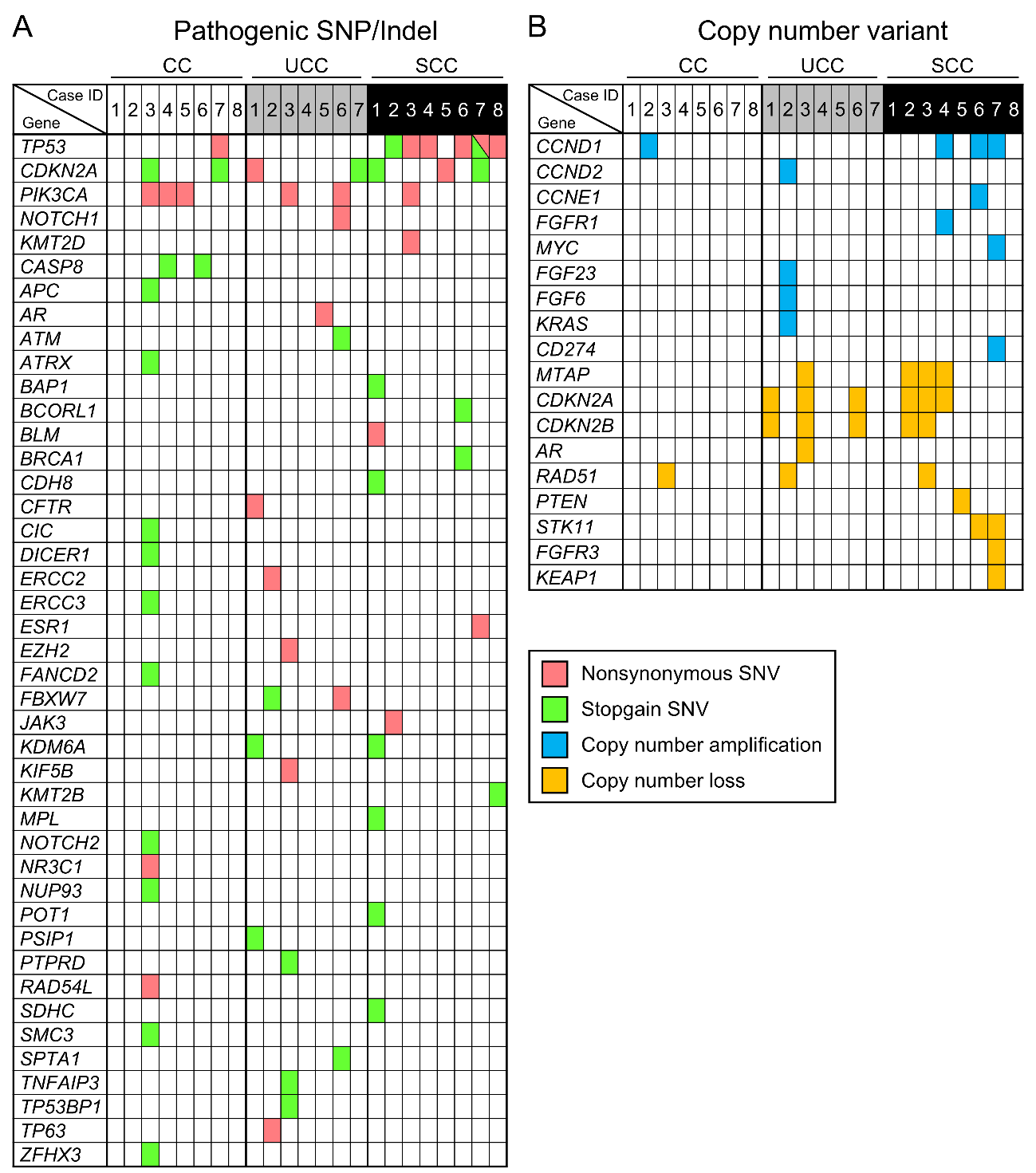


**Supplementary Fig. 2.** Summary of the genetic alterations of the CC, UCC, and SCC groups. **A:** Summary of pathogenic gene mutations (SNVs or Indels) among the CC, UCC, and SCC groups. **B:** Summary of CNVs in the three groups. *CC* carcinoma cuniculatum, *CNV* copy number variation, *Indel* insertion/deletion, *SCC* squamous cell carcinoma, *SNV* single-nucleotide variant, *UCC* uncertain carcinoma cuniculatum
